# An uncommon encounter with tumor lysis syndrome in colorectal cancer

**DOI:** 10.3205/000361

**Published:** 2026-06-18

**Authors:** Ursula M. A. de Matos, Wan Ying Tan, Moana Divina Da Silva Santiago, Qian Wu, Faripour Forouhar, Ritika Vankina, Pragna Kapadia, Victoria Forbes

**Affiliations:** 1 Department of Internal Medicine, School of Medicine, University of Connecticut, Farmington, CT, USA; 2 Department of Hematology and Oncology, Carole and Ray Neag Comprehensive Cancer Center, University of Connecticut Health Center, Farmington, CT, USA; 3 Surgical Oncology Research Laboratories, Department of Surgery, Yale School of Medicine, New Haven, CT, USA; 4 Department of Pathology, University of Connecticut School of Medicine, Farmington, CT, USA

## Abstract

Tumor lysis syndrome (TLS) is a rare but life-threatening complication in solid tumors such as colorectal cancer (CRC). We report a 61-year-old male with metastatic CRC and extensive liver metastases who developed acute TLS within 48 hours of initiating FOLFOX chemotherapy. He presented with acute kidney injury (AKI), hyperuricemia, hyperphosphatemia, hypocalcemia, and elevated liver enzymes. Prompt treatment with intravenous fluids, rasburicase, and febuxostat led to rapid resolution of TLS. Despite recovery, the patient died two weeks later from complications of advanced metastatic disease. This case highlights the need for early TLS recognition in high-risk CRC patients.

## Introduction

Tumor lysis syndrome (TLS) is an oncologic emergency resulting from the rapid destruction of malignant cells, leading to characteristic metabolic abnormalities, including hyperkalemia, hyperuricemia, hyperphosphatemia, and hypocalcemia. TLS may be diagnosed based on laboratory criteria alone or as clinical TLS when accompanied by complications such as acute kidney injury (AKI), arrhythmias, seizures, or sudden cardiac death [1].

While TLS is more commonly associated with hematologic malignancies, it can also rarely occur in solid tumors. Tumor-specific risk factors for TLS include proliferation rate, sensitivity to treatment, and tumor burden [2], [3]. TLS most frequently arises after chemotherapy but can also develop following localized radiation, metastasis ablation, or even in the absence of treatment [4]. Clinically, TLS can present with AKI due to uric acid and calcium phosphate crystal precipitation in renal tubules, as well as cardiac arrhythmias and seizures caused by electrolyte disturbances. 

We report a rare case of TLS in a patient with metastatic colorectal cancer following FOLFOX chemotherapy. This case highlights a potentially under-recognized complication of a standard first-line regimen and adds to growing evidence that TLS in colorectal cancer (CRC), though uncommon, is associated with significant mortality. We aim to expand awareness, emphasize the importance of early recognition, and support the need for updated risk stratification in patients with advanced CRC.

## Case description

**Figure 1:**
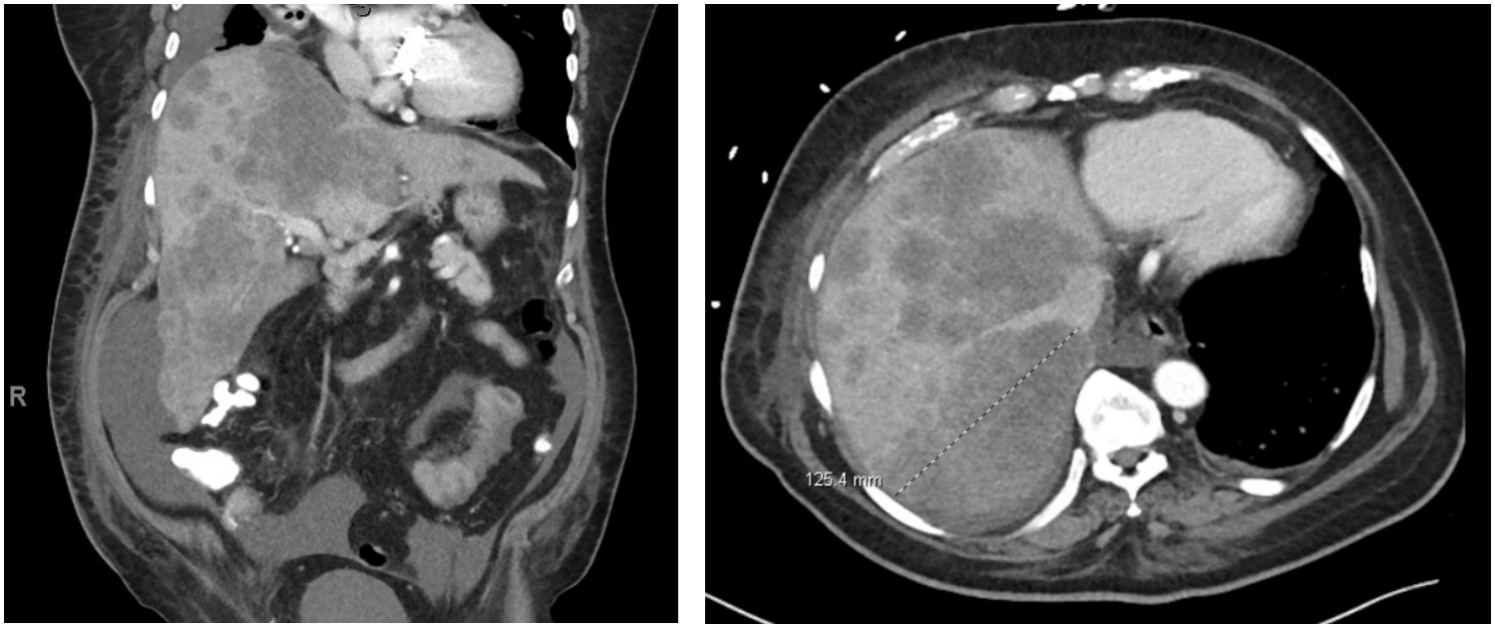
Coronal (left) and cross-sectional (right) view of CT chest/abdomen/pelvis

A 61-year-old male with past medical history notable for mitral and aortic valve endocarditis status post replacement and renal cell carcinoma status post left nephrectomy who initially presented for subacute cough. Initial chest CT showed no pulmonary masses but incidentally revealed multiple low-density liver lesions. A subsequent CT of the abdomen and pelvis confirmed the presence of numerous large, ill-defined hepatic lesions, consistent with metastatic disease, as well as a 1.5-cm hypodensity in the proximal right portal vein concerning for thrombosis (Figure 1 (Fig. 1)).

**Figure 2:**
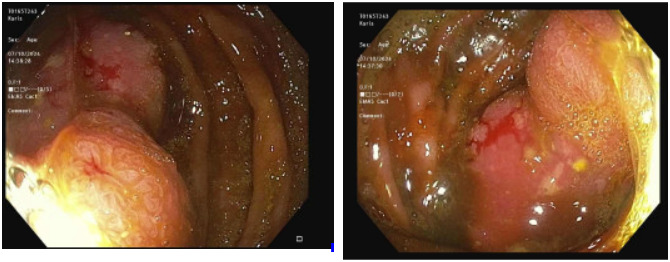
Intraluminal mass at the cecum identified through colonoscopy

**Figure 3:**
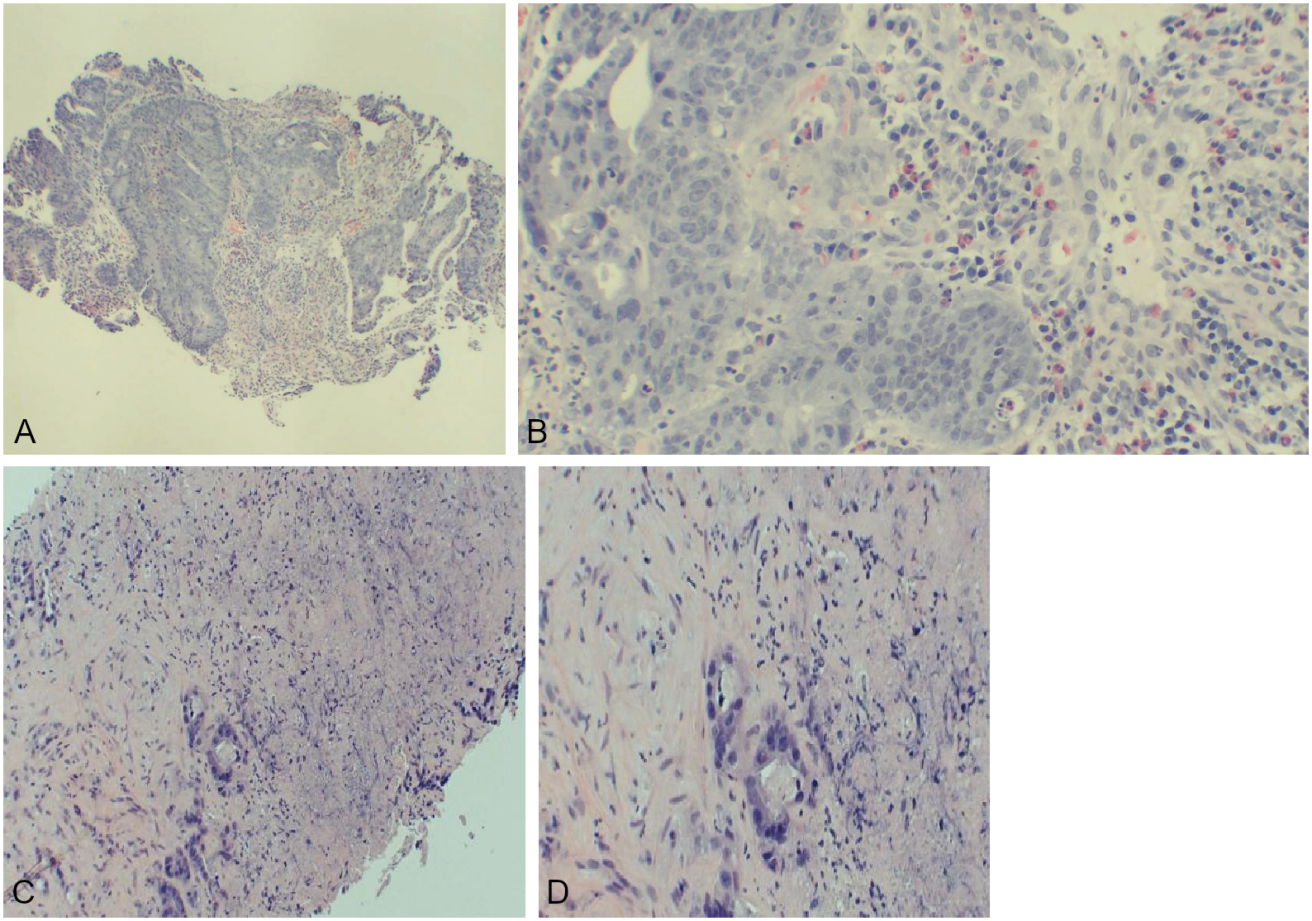
H&E stain of cecal biopsy (A and B) and liver biopsy (C and D) that shows nest of adenocarcinoma

Colonoscopy revealed a polypoid mass in the cecum (Figure 2 (Fig. 2)) and biopsy confirmed the diagnosis of colon adenocarcinoma with TP53 mutated disease, KRAS G12d, high tumor mutation burden, APC and PIK3CA mutated, with intact mismatch repair (MMR) proteins and no loss of nuclear expression, indicating a low probability of microsatellite instability-high (MSI-H) status (Figure 3 (Fig. 3)).

The patient was placed on a planned 48-hour infusion pump of 5-fluorouracil (5-FU), leucovorin, and oxaliplatin. The following day, the pump was found disconnected, and he presented hypotensive and tachycardic to the infusion clinic, prompting transfer to the emergency department. Initial investigation revealed significant laboratory abnormalities (Table 1 (Tab. 1)), including AST 293 U/L (previously 136 U/L), ALT 133 U/L (previously 105 U/L), alkaline phosphatase 1,053 U/L (previously 249 U/L), total bilirubin 1.9 mg/dL (previously normal), lactic acid level 4.2 mmol/L, and white blood cell count of 18.6×10^9^/L. Notably, uric acid levels were elevated at 18.7 mg/dL, phosphate 6.1 mg/dL, calcium 6.9 mg/dL, and creatinine of 2.5 mg/dL (elevated from baseline of 1 mg/dL), meeting criteria for clinical TLS.

Treatment was initiated with rasburicase, intravenous fluids, and febuxostat, resulting in TLS resolution within four days. Two weeks after discharge, he was rehospitalized for hypoxia due to pneumonia and malignant pleural effusion and died despite aggressive treatment. 

## Discussion

TLS is rare in solid tumors, particularly in CRC. The first documented case of TLS in a non-hematologic malignancy was reported in 1977, involving a patient with widespread adenocarcinoma of gastrointestinal origin without prior chemotherapy [5]. A 2012 review identified only 100 cases of TLS in solid tumors from 1977 to 2011, indicating that the primary risk factors for TLS in solid tumors are large tumor burden and liver metastasis, with 83% of patients exhibiting metastatic disease, most commonly in the liver [6]. A 2020 review found 9 cases of CRC related TLS, all of which were metastatic, with liver involvement in 80% of cases [7]. It is unclear whether the association between liver metastasis in solid tumor associated TLS reflects increased tumor burden or is an independent risk factor related to reduced purine and uric acid metabolism [6]. Notably, our patient also presented with portal vein thrombosis (PVT) and five other cases in the literature were found linking solid tumor-associated TLS with PVT [3], [4], [8], [9], [10], [11]. These reports allow us to speculate that PVT may exacerbate TLS by promoting ischemia-induced cell lysis and impairing hepatic clearance of TLS-related byproducts [3]. 

Other risk factors include an elevated lactate dehydrogenase level and hyperuricemia before therapy initiation. Furthermore, preexisting renal dysfunction already poses higher risk. In our patient with a solitary kidney and no known history of chronic kidney disease, it is important to recognize that a single kidney has reduced renal reserve despite normal baseline function. This limits the kidney’s ability to handle sudden metabolic stress and decreases its tolerance to insults such as dehydration, hypotension, or exposure to nephrotoxic medications, and could have been a contributing risk factor for the development of TLS.

Previously, TLS was not associated with CRC, but with the advent of more aggressive therapies, the incidence appears to be increasing [7], [12], [13]. This trend is significant given the high mortality associated with TLS in solid tumors, ranging from 35% to 60%, compared to 21% for overall in-hospital TLS mortality [7], [14], [15], [16] and rates as low as 2% in hematologic malignancies when managed preventively [17], [18]. It remains uncertain whether TLS in solid tumors is intrinsically more aggressive or whether the higher mortality reflects patient and disease-related factors, as these patients have substantial tumor burden and often experience poorer overall health and cachexia, multi-organ involvement or inherently more aggressive cancer subtypes. Additionally, limited awareness among healthcare providers regarding the occurrence of TLS in CRC may delay diagnosis and life-saving interventions, such as urate oxidase administration, aggressive intravenous hydration, and allopurinol.

## Conclusion

This case stresses the importance of recognizing TLS as a potential complication in CRC patients, especially those who undergo chemotherapy with high disease burden and liver metastasis. As novel therapies for solid tumors emerge, vigilance for TLS is essential due to its high mortality. Future research is needed to identify biomarkers and molecular mutations associated with an increased risk of TLS. To date, no molecular mutations have been definitively linked to increased TLS risk in CRC, highlighting a potential area for further investigation. 

## Notes

### Competing interests

The authors declare that they have no competing interests.

## Figures and Tables

**Table 1:**
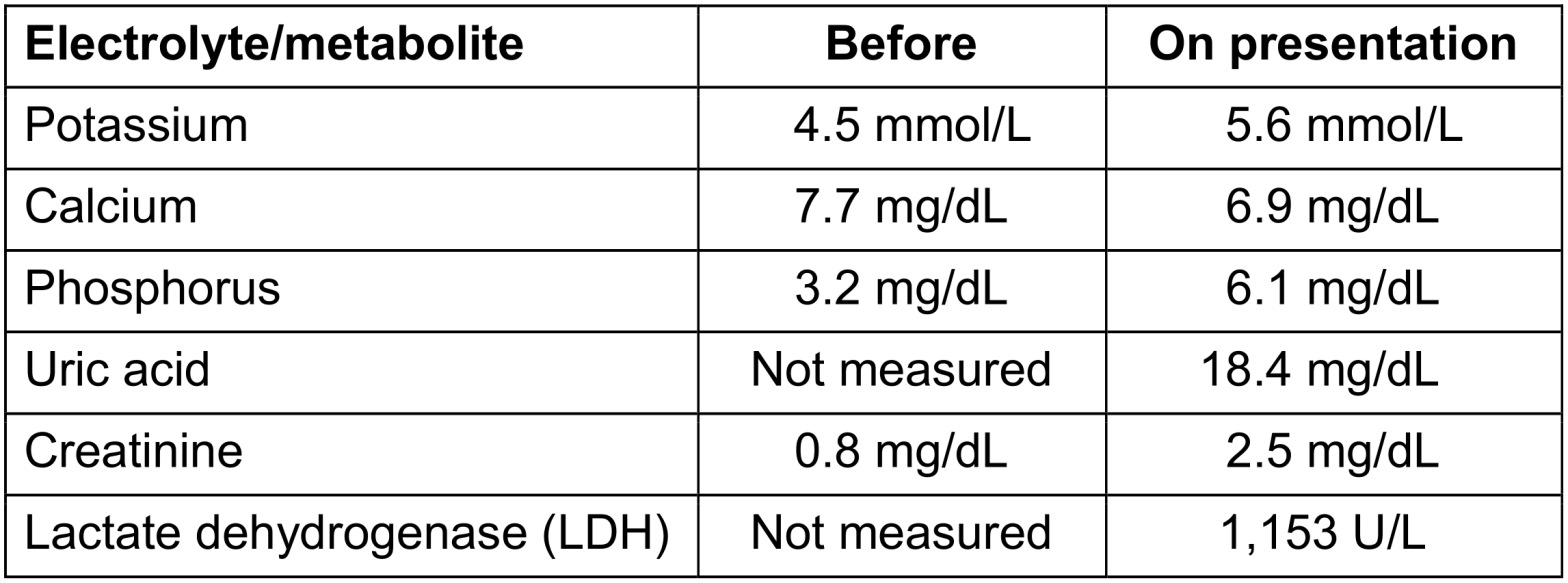
Levels of electrolytes and metabolites before and on presentation
